# 2-(Pyridin-2-yl)-1,3-oxathiane

**DOI:** 10.1107/S1600536812018661

**Published:** 2012-05-12

**Authors:** David Turner, Albert Fratini, Claudia Turro, Michael Check, Chad Hunter

**Affiliations:** a Thermal Sciences and Materials Branch, Material and Manufacturing Directorate, Air Force Research Laboratory, Wright-Patterson Air Force Base, OH 45433, USA; b Universal Technology Corporation, 1270 N. Fairfield Road, Beavercreek, OH 45432, USA; c Chemistry Department, University of Dayton, 300 College Park, Dayton, OH 45469-2357, USA; d Chemistry Department, The Ohio State University, 154 W. 12th Avenue, Columbus, OH 43210, USA

## Abstract

The title compound, C_9_H_11_NOS, exhibits a unique structural motif, with free rotation of the aliphatic oxathiane ring about the C—C bond connecting this moiety to the aromatic pyridine ring. The structure elucidation was undertaken due to its potential as a bidentate ligand for organometallic complexes. The oxathiane ring adopts the expected chair conformation, with the S atom in proximity to the N atom on the pyridine ring. The corresponding S—C—C—N torsion angle is 69.07 (14)°. In the crystal, mol­ecules aggregate as centrosymmetric pairs connected by pairs of C—H⋯N hydrogen bonds.

## Related literature

The corresponding organic compound, 2-(2-pyridyl)-1,3-oxathiane, forms dimers *via* weak inter­molecular C—H⋯N hydrogen bonds, exhibiting similar photophysical properties as previously observed (Rachford *et al.*, 2005 ▶; Rachford & Rack, 2006 ▶).
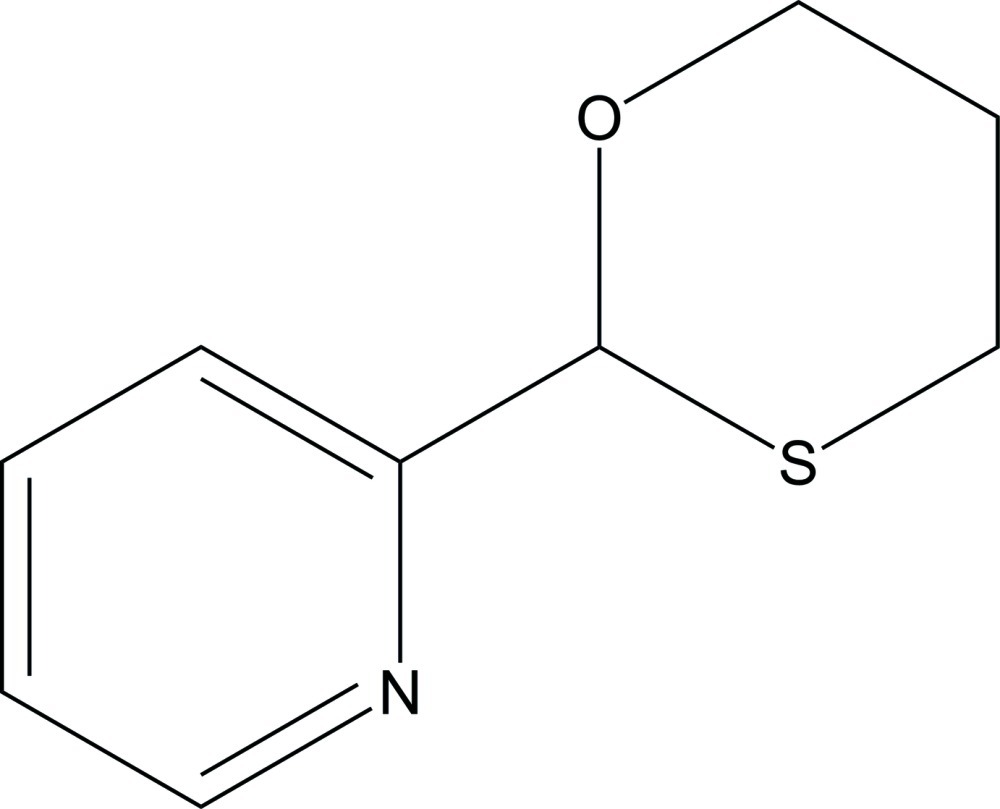


## Experimental

### 

#### Crystal data

C_9_H_11_NOS
*M* *_r_* = 181.26
Monoclinic, 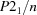

*a* = 7.5329 (3) Å
*b* = 11.8099 (5) Å
*c* = 9.7632 (4) Å
β = 92.940 (3)°
*V* = 867.42 (6) Å^3^
*Z* = 4
Cu *K*α radiation
μ = 2.89 mm^−1^
*T* = 110 K
0.48 × 0.46 × 0.36 mm

#### Data collection

Oxford Diffraction Xcalibur Sapphire3 diffractometer
Absorption correction: analytical [*CrysAlis PRO* (Oxford Diffraction, 2010 ▶), based on expressions derived by Clark & Reid (1995 ▶)] *T*_min_ = 0.344, *T*_max_ = 0.519
3675 measured reflections
1708 independent reflections
1656 reflections with *I* > 2σ(*I*)
*R*_int_ = 0.020

#### Refinement

*R*[*F* ^2^ > 2σ(*F* ^2^)] = 0.032
*wR*(*F* ^2^) = 0.085
*S* = 1.07
1708 reflections
154 parameters
All H-atom parameters refined
Δρ_max_ = 0.32 e Å^−3^
Δρ_min_ = −0.30 e Å^−3^

### 

Data collection: *CrysAlis PRO* (Oxford Diffraction, 2010 ▶); cell refinement: *CrysAlis PRO*; data reduction: *CrysAlis PRO*; program(s) used to solve structure: *SHELXS97* (Sheldrick, 2008 ▶); program(s) used to refine structure: *SHELXL97* (Sheldrick, 2008) ▶; molecular graphics: *ORTEP-3 for Windows* (Farrugia, 1997 ▶); software used to prepare material for publication: *SHELXTL* (Sheldrick, 2008 ▶).

## Supplementary Material

Crystal structure: contains datablock(s) I, global. DOI: 10.1107/S1600536812018661/nr2024sup1.cif

Structure factors: contains datablock(s) I. DOI: 10.1107/S1600536812018661/nr2024Isup2.hkl

Supplementary material file. DOI: 10.1107/S1600536812018661/nr2024Isup3.cml

Additional supplementary materials:  crystallographic information; 3D view; checkCIF report

## Figures and Tables

**Table 1:** Hydrogen-bond geometry (Å, °)

*D*—H⋯*A*	*D*—H	H⋯*A*	*D*⋯*A*	*D*—H⋯*A*
C1—H1⋯N1^i^	0.979 (19)	2.586 (19)	3.5399 (19)	164.8 (14)

Symmetry code: (i) 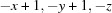.

**Fig. 1.**
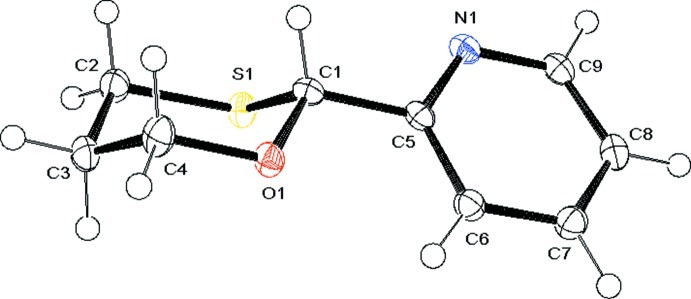
The molecular structure of the title compound. Thermal ellipsoids are drawn at 50% probability for non-H atoms.

**Fig. 2.**
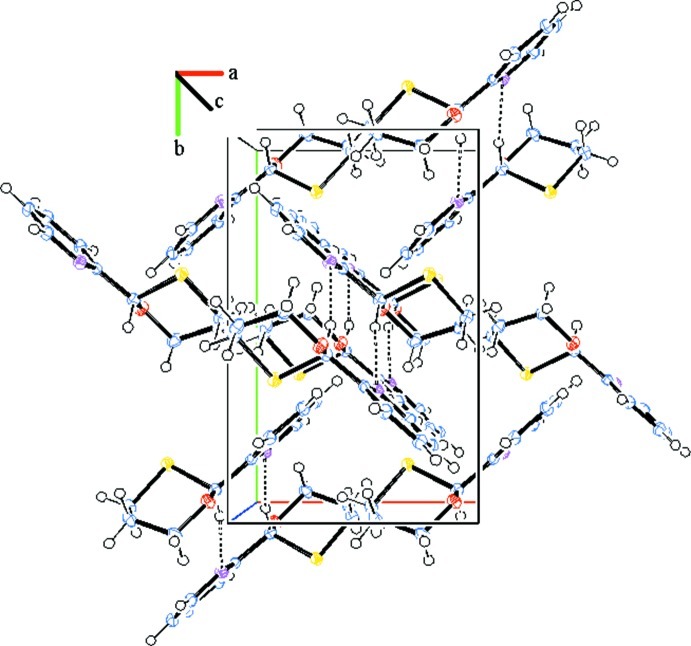
The crystal packing plot of the title compound viewed down the c-axis. C1—H1···N1 hydrogen bonds are drawn as dashed lines.

## supplementary crystallographic information

### Comment

Photo-induced or photo-triggered molecular isomerizations employ the stored energy in an electronic excited state for rapid bond-breaking and bond-making reactions. One of the most well studied examples of this type of reaction is photo-isomerization of stilbene and its derivatives, where phenyl group rotation occurs following π→π^*^ excitation on an ultrafast time scale. Photo-induced or photo-triggered linkage isomerizations have also been observed in certain late transition metal complexes containing NO^+^, NO^2-^, N_2_, SO_2_, and DMSO (dimethylsulfoxide). Rack *et al.* has worked on ruthenium complexes with DMSO ligands and has observed photo- isomerization between the S-bound to the O-bound state upon uv/visible irradiation. However, this conversion can only be demonstrated in a solvent of DMSO (Rachford *et al.*, 2005; Rachford & Rack, 2006). The development of photo-switchable molecules is of interest due to potential use in applications such as optical molecular information storage, optical limiting devices, and molecular sensing. For photonic devices, the design of such molecules requires the efficient conversion of light energy to potential energy. Thus, bistable molecules are also of a fundamental interest in that the design of such molecules requires specific electronic structures in order to exhibit two stable interconvertible states. Rack *et al.* has worked on ruthenium complexes with DMSO ligands and has observed photo-isomerization between the S-bound to the O-bound state upon uv/visible irradiation. However, this conversion can only be demonstrated in a solvent of DMSO (Rachford *et al.*, 2005; Rachford & Rack, 2006). The synthesis and bonding of 2-(2-pyridyl)-1,3-oxathiane to a ruthenium metal center would still allow for the photo-isomerization between a S-bound to an O-bound state upon uv/visible irradiation due to the ability of the bidentate ligand to rotate about the C—C bond between the aliphatic, oxathiane moiety and the aromatic, pyridyl moiety. The major benefit of using this bidentate ligand would be that the photo-isomerization could be performed in a wide variety of solvents.

### Experimental

The title compound was synthesized as follows: A solution of 3.34 g (31.2 mmol) of 2-pyridinecarbaldehyde, 10.0 g (109 mmol) of 3-mercapto-1-propanol, and 0.475 g (2.50 mmol) of *p*-toluenesulfonic acid monohydrate in 400 ml of 1,2-dichloroethane were refluxed for 24 h with a Dean-Stark trap to collect the azeotroped water. After cooling, the azeotroped water was disposed of. The reacted mixture was washed with 70 ml of 7 *M* KOH and water. The aqueous and organic layers were separated in a separatory funnel. The organic layer was then dried over anhydrous sodium sulfate and filtered to remove the Na_2_SO_4_. The resulting solution was evaporated under reduced pressure to yield a brown oil. The brown oil was then passed through a silica column with diethyl ether. The 2-(2-pyridyl)-1,3-oxathiane was collected from the column and dried in air with a yield of 4.29 g (76%): ^1^H-NMR (400 MHz Bruker, CDCl_3_) δ(p.p.m.) 1.58 (d, 1 H), 1.92 (dd, 1 H), 2.68 (d, 1 H), 3.05 (dd, 1 H), 3.64 (dd, 1 H), 4.17 (d, 1 H), 5.80 (s, 1 H), 7.04 (t, 1 H), 7.41 (d, 1 H), 7.54 (t, 1 H), 8.41 (d, 1 H). ^13^C-NMR (400 MHz Bruker, CDCl_3_) δ(p.p.m.) 24.8 (CH_2_), 27.9 (CH_2_), 69.6 (CH_2_), 84.3 (CH), 120.1 (CH), 122.4 (CH), 136.0 (CH), 148.0 (CH), 157.2 (C). The experimental protocol for recrystallizing the title compound was as follows: 100 mg of 2-(2-pyridyl)-1,3-oxathiane was dissolved in 0.5 ml of methylene chloride, followed by the addition of 2.0 ml of hexane to the solution. The solution was filtered and then placed in a vial, covered with parafilm, and allowed to evaporate at room temperature over the course of days, after which time large crystals were obtained.

### Refinement

All non-hydrogen atoms were refined anistropically. All H-atoms were located in difference maps and refined free with isotropic displacement parameters.

### Crystal data

**Table:**

C_9_H_11_NOS	*F*(000) = 384
*M**_r_* = 181.26	*D*_x_ = 1.388 Mg m^−^^3^
Monoclinic, *P*2_1_/*n*	Cu *K*α radiation, λ = 1.54178 Å
Hall symbol: -P 2yn	Cell parameters from 3102 reflections
*a* = 7.5329 (3) Å	θ = 0.5–72.0°
*b* = 11.8099 (5) Å	µ = 2.89 mm^−^^1^
*c* = 9.7632 (4) Å	*T* = 110 K
β = 92.940 (3)°	Block, colourless
*V* = 867.42 (6) Å^3^	0.48 × 0.46 × 0.36 mm
*Z* = 4	

### Data collection

**Table:**

Oxford Diffraction Xcalibur Sapphire3 diffractometer	1708 independent reflections
Radiation source: Enhance (Cu) xray source	1656 reflections with *I* > 2σ(*I*)
Graphite monochromator	*R*_int_ = 0.020
Detector resolution: 16.3384 pixels mm^-1^	θ_max_ = 72.1°, θ_min_ = 5.9°
ω scans	*h* = −6→9
Absorption correction: analytical [*CrysAlis PRO* (Oxford Diffraction, 2010), based on expressions derived by Clark & Reid (1995)]	*k* = −12→14
*T*_min_ = 0.344, *T*_max_ = 0.519	*l* = −12→12
3675 measured reflections	

### Refinement

**Table:**

Refinement on *F*^2^	Secondary atom site location: difference Fourier map
Least-squares matrix: full	Hydrogen site location: inferred from neighbouring sites
*R*[*F*^2^ > 2σ(*F*^2^)] = 0.032	All H-atom parameters refined
*wR*(*F*^2^) = 0.085	*w* = 1/[σ^2^(*F*_o_^2^) + (0.0498*P*)^2^ + 0.3816*P*] where *P* = (*F*_o_^2^ + 2*F*_c_^2^)/3
*S* = 1.07	(Δ/σ)_max_ < 0.001
1708 reflections	Δρ_max_ = 0.32 e Å^−^^3^
154 parameters	Δρ_min_ = −0.30 e Å^−^^3^
0 restraints	Extinction correction: *SHELXL97* (Sheldrick, 2008), Fc^*^=kFc[1+0.001xFc^2^λ^3^/sin(2θ)]^-1/4^
Primary atom site location: structure-invariant direct methods	Extinction coefficient: 0.0187 (14)

### Special details

**Table:**

Experimental. *CrysAlis PRO* (Oxford Diffraction, 2010). Analytical numeric absorption correction using a multifaceted crystal model based on expressions derived by *R*. C. Clark & J. S. Reid (Clark & Reid, 1995).
Geometry. All e.s.d.'s (except the e.s.d. in the dihedral angle between two l.s. planes) are estimated using the full covariance matrix. The cell e.s.d.'s are taken into account individually in the estimation of e.s.d.'s in distances, angles and torsion angles; correlations between e.s.d.'s in cell parameters are only used when they are defined by crystal symmetry. An approximate (isotropic) treatment of cell e.s.d.'s is used for estimating e.s.d.'s involving l.s. planes.
Refinement. Refinement of *F*^2^ against ALL reflections. The weighted *R*-factor *wR* and goodness of fit *S* are based on *F*^2^, conventional *R*-factors *R* are based on *F*, with *F* set to zero for negative *F*^2^. The threshold expression of *F*^2^ > σ(*F*^2^) is used only for calculating *R*-factors(gt) *etc*. and is not relevant to the choice of reflections for refinement. *R*-factors based on *F*^2^ are statistically about twice as large as those based on *F*, and *R*- factors based on ALL data will be even larger.

### Fractional atomic coordinates and isotropic or equivalent isotropic displacement parameters (Å^2^)

**Table:**

	*x*	*y*	*z*	*U*_iso_*/*U*_eq_	
S1	0.20151 (4)	0.36641 (3)	0.12188 (3)	0.01796 (16)	
O1	0.39908 (13)	0.45308 (9)	0.33518 (10)	0.0168 (2)	
N1	0.59533 (16)	0.33730 (11)	0.03808 (12)	0.0174 (3)	
C1	0.40671 (19)	0.43156 (12)	0.19284 (14)	0.0153 (3)	
H1	0.421 (2)	0.5024 (17)	0.1426 (18)	0.020 (4)*	
C2	0.05140 (19)	0.47642 (13)	0.17565 (15)	0.0190 (3)	
H2A	0.070 (3)	0.5468 (17)	0.1214 (19)	0.025 (5)*	
H2B	−0.069 (3)	0.4511 (17)	0.1510 (19)	0.023 (5)*	
C3	0.0792 (2)	0.49944 (14)	0.32842 (15)	0.0202 (3)	
H3B	0.002 (3)	0.5605 (18)	0.354 (2)	0.032 (5)*	
H3A	0.049 (3)	0.4302 (18)	0.381 (2)	0.029 (5)*	
C4	0.2682 (2)	0.53679 (13)	0.36591 (16)	0.0209 (3)	
H4A	0.295 (3)	0.6069 (18)	0.316 (2)	0.028 (5)*	
H4B	0.283 (2)	0.5485 (16)	0.465 (2)	0.022 (5)*	
C5	0.55562 (19)	0.35019 (12)	0.16969 (14)	0.0150 (3)	
C6	0.64241 (19)	0.29098 (12)	0.27685 (15)	0.0173 (3)	
H6	0.607 (3)	0.3005 (17)	0.368 (2)	0.025 (5)*	
C7	0.7761 (2)	0.21501 (13)	0.24709 (16)	0.0196 (3)	
H7	0.839 (3)	0.1741 (17)	0.322 (2)	0.024 (5)*	
C8	0.81619 (19)	0.19959 (13)	0.11104 (16)	0.0192 (3)	
H8	0.903 (3)	0.1456 (16)	0.089 (2)	0.023 (5)*	
C9	0.72283 (19)	0.26255 (13)	0.01104 (15)	0.0182 (3)	
H9	0.751 (2)	0.2565 (16)	−0.082 (2)	0.018 (4)*	

### Atomic displacement parameters (Å^2^)

**Table:**

	*U*^11^	*U*^22^	*U*^33^	*U*^12^	*U*^13^	*U*^23^
S1	0.0163 (2)	0.0193 (2)	0.0182 (2)	0.00117 (12)	−0.00020 (14)	−0.00488 (12)
O1	0.0182 (5)	0.0197 (5)	0.0127 (5)	0.0034 (4)	0.0013 (4)	−0.0034 (4)
N1	0.0188 (6)	0.0182 (6)	0.0155 (6)	−0.0010 (5)	0.0038 (5)	−0.0002 (5)
C1	0.0175 (7)	0.0164 (7)	0.0120 (6)	−0.0013 (5)	0.0021 (5)	0.0006 (5)
C2	0.0171 (7)	0.0217 (7)	0.0183 (7)	0.0028 (6)	0.0006 (5)	0.0000 (6)
C3	0.0200 (7)	0.0224 (8)	0.0188 (7)	0.0057 (6)	0.0047 (5)	−0.0012 (6)
C4	0.0231 (7)	0.0193 (7)	0.0202 (7)	0.0046 (6)	0.0008 (6)	−0.0069 (6)
C5	0.0147 (7)	0.0149 (6)	0.0156 (7)	−0.0033 (5)	0.0033 (5)	−0.0009 (5)
C6	0.0167 (7)	0.0198 (7)	0.0154 (7)	−0.0004 (5)	0.0015 (5)	0.0002 (5)
C7	0.0184 (7)	0.0200 (7)	0.0202 (7)	0.0006 (6)	−0.0003 (6)	0.0011 (6)
C8	0.0151 (7)	0.0193 (7)	0.0234 (8)	−0.0001 (6)	0.0036 (6)	−0.0029 (6)
C9	0.0187 (7)	0.0201 (7)	0.0163 (7)	−0.0020 (6)	0.0050 (5)	−0.0020 (6)

### Geometric parameters (Å, º)

**Table:**

S1—C2	1.8174 (15)	C3—H3B	0.97 (2)
S1—C1	1.8307 (14)	C3—H3A	1.00 (2)
O1—C1	1.4168 (16)	C4—H4A	0.99 (2)
O1—C4	1.4386 (17)	C4—H4B	0.973 (19)
N1—C9	1.3407 (19)	C5—C6	1.393 (2)
N1—C5	1.3428 (18)	C6—C7	1.390 (2)
C1—C5	1.5030 (19)	C6—H6	0.95 (2)
C1—H1	0.979 (19)	C7—C8	1.389 (2)
C2—C3	1.520 (2)	C7—H7	0.98 (2)
C2—H2A	1.00 (2)	C8—C9	1.389 (2)
C2—H2B	0.97 (2)	C8—H8	0.95 (2)
C3—C4	1.517 (2)	C9—H9	0.951 (19)
			
C2—S1—C1	96.66 (7)	O1—C4—C3	113.23 (12)
C1—O1—C4	112.99 (11)	O1—C4—H4A	108.2 (12)
C9—N1—C5	117.39 (13)	C3—C4—H4A	109.6 (12)
O1—C1—C5	109.29 (11)	O1—C4—H4B	105.1 (11)
O1—C1—S1	111.76 (9)	C3—C4—H4B	109.6 (11)
C5—C1—S1	107.25 (10)	H4A—C4—H4B	111.0 (16)
O1—C1—H1	110.5 (11)	N1—C5—C6	122.87 (13)
C5—C1—H1	111.6 (11)	N1—C5—C1	114.90 (12)
S1—C1—H1	106.5 (11)	C6—C5—C1	122.22 (13)
C3—C2—S1	110.78 (10)	C7—C6—C5	118.94 (14)
C3—C2—H2A	110.8 (11)	C7—C6—H6	120.8 (12)
S1—C2—H2A	109.6 (11)	C5—C6—H6	120.2 (12)
C3—C2—H2B	112.2 (11)	C8—C7—C6	118.67 (14)
S1—C2—H2B	107.1 (12)	C8—C7—H7	122.0 (12)
H2A—C2—H2B	106.2 (16)	C6—C7—H7	119.3 (12)
C4—C3—C2	111.67 (12)	C7—C8—C9	118.34 (14)
C4—C3—H3B	106.9 (12)	C7—C8—H8	119.3 (12)
C2—C3—H3B	109.6 (12)	C9—C8—H8	122.4 (12)
C4—C3—H3A	110.4 (12)	N1—C9—C8	123.77 (13)
C2—C3—H3A	109.6 (12)	N1—C9—H9	115.9 (11)
H3B—C3—H3A	108.6 (16)	C8—C9—H9	120.3 (11)
			
C4—O1—C1—C5	175.71 (11)	O1—C1—C5—N1	−169.60 (11)
C4—O1—C1—S1	−65.74 (13)	S1—C1—C5—N1	69.07 (14)
C2—S1—C1—O1	56.31 (11)	O1—C1—C5—C6	11.82 (18)
C2—S1—C1—C5	176.07 (9)	S1—C1—C5—C6	−109.50 (13)
C1—S1—C2—C3	−52.99 (12)	N1—C5—C6—C7	0.0 (2)
S1—C2—C3—C4	59.31 (15)	C1—C5—C6—C7	178.42 (13)
C1—O1—C4—C3	64.95 (16)	C5—C6—C7—C8	−1.0 (2)
C2—C3—C4—O1	−61.17 (17)	C6—C7—C8—C9	1.2 (2)
C9—N1—C5—C6	0.9 (2)	C5—N1—C9—C8	−0.8 (2)
C9—N1—C5—C1	−177.62 (12)	C7—C8—C9—N1	−0.3 (2)

### Hydrogen-bond geometry (Å, º)

**Table:**

*D*—H···*A*	*D*—H	H···*A*	*D*···*A*	*D*—H···*A*
C1—H1···N1^i^	0.979 (19)	2.586 (19)	3.5399 (19)	164.8 (14)

Symmetry code: (i) −*x*+1, −*y*+1, −*z*.
